# THC-induced behavioral stereotypy in zebrafish as a model of psychosis-like behavior

**DOI:** 10.1038/s41598-021-95016-4

**Published:** 2021-08-03

**Authors:** Amelia Dahlén, Mahdi Zarei, Adam Melgoza, Mahendra Wagle, Su Guo

**Affiliations:** 1 Department of Bioengineering and Therapeutic Sciences, and Programs in Biological Sciences and Human Genetics, University of California, San Francisco, CA 94158 USA; 2 Section of Functional Pharmacology, Department of Neuroscience, Uppsala University, 75124 Uppsala, Sweden

**Keywords:** Neuroscience, Pharmacology

## Abstract

High doses of the *Cannabis* constituent Δ9-tetrahydrocannabinol (THC) increase the risk of psychosis in humans. Highly accessible animal models are needed to address underlying mechanisms. Using zebrafish with a conserved endocannabinoid system, this study investigates the acute effects of THC on adult zebrafish behavior and the mechanisms involved. A concentration-dependent THC-induced behavioral stereotypy akin to THC’s effect in rats and the psychotropics phencyclidine and ketamine in zebrafish was established. Distinctive circular swimming during THC-exposure was measured using a novel analytical method that we developed, which detected an elevated Repetition Index (RI) compared to vehicle controls. This was reduced upon co-administration of N-methyl-D-aspartate (NMDA) receptor agonist NMDA, suggesting that THC exerts its effects via biochemical or neurobiological mechanisms associated with NMDA receptor antagonism. Co-treatment of γ‐aminobutyric acid receptor antagonist pentylenetetrazol also showed signs of reducing the RI. Since THC-induced repetitive behavior remained in co-administrations with cannabinoid receptor 1 inverse agonist AM251, the phenotype may be cannabinoid receptor 1-independent. Conversely, the inverse cannabinoid receptor 2 agonist AM630 significantly reduced THC-induced behavioral stereotypy, indicating cannabinoid receptor 2 as a possible mediator. A significant reduction of the THC-RI was also observed by the antipsychotic sulpiride. Together, these findings highlight this model’s potential for elucidating the mechanistic relationship between *Cannabis* and psychosis.

## Introduction

*Cannabis*, a substance derived from the *Cannabis indica* and *Cannabis sativa* plants, has a wide array of both beneficial and harmful properties which makes its use a controversial topic^1^. Medicinally, *Cannabis* acts as an analgesic^2^, anti-emetic^3^ and appetite stimulant^4^. Recreationally, it is an anxiolytic producing a sense of euphoria^5^. However, *Cannabis* has also been identified as a risk factor for inducing acute psychoses in healthy individuals^6,7^ and schizophrenia in individuals susceptible to mental illness^7–9^. Schizophrenia is a chronic mental disorder with cognitive, emotional and behavioral disturbances, affecting ~1% of the global population^10^. The complex psychiatric condition is manifested through an array of negative symptoms, such as anhedonia and alogia, as well as through positive symptoms like disordered thoughts and catatonia^11^. Psychosis, comprising of episodic delusions and hallucinations, is an additional symptom of schizophrenia and may also be brought on by illness, extreme stress or drug use^11–13^.

Drugs that trigger psychotic symptoms in humans, such as the hallucinogenic phencyclidine (PCP) and the sedative ketamine^14,15^, have been found to initiate repetitive stereotyped circling when administered to zebrafish (*Danio rerio*)^16,17^. Interestingly, rotational swimming has not been observed following administration of other psychotropics like lysergic acid (LSD), 3,4,5-Trimethoxyphenethylamine (mescaline) or 3,4-Methylenedioxymethamphetamine (MDMA)^16,18,19^. Consequently, this distinct behavioral stereotypy has been attributed to a mechanism shared between PCP and ketamine, namely antagonism of the glutamate N-methyl-D-aspartate receptor (NMDAR)^20^. Glutamate is the predominant excitatory neurotransmitter in the central nervous system (CNS) and acts as a precursor to the main inhibitory neurotransmitter γ‐aminobutyric acid (GABA)^21^. Together they work to maintain an excitation/inhibition balance. Inhibition of the ionotropic NMDAR impedes further excitatory signaling and may give rise to the repetitive stereotyped circling^22^. This NMDAR hypofunction is also a prominent clinical hallmark of psychosis and schizophrenia, and thus the animal behavior stereotypy has potential as a measure of psychosis-like behavior^23^. Hereafter, the psychosis-like behavior refers to the circling behavior as a psychopharmacological response relevant to human psychosis.

Similar to PCP and ketamine, the main psychoactive component of *Cannabis*, Δ9-tetrahydrocannabinol (THC), causes circling in rats^24,25^. THC binds G_i/o_-protein coupled cannabinoid receptors in the brain (CB_1_R) and periphery (CB_2_R)^26^, although CB_2_R expression has also been reported in the midbrain dopamine neurons^27^. Since administration of the CB_1_R antagonist SR-141716 eliminates the THC-induced circling in rats, the behavior is hypothesized to be mediated through CB_1_R^24^. Among a broad range of downstream effects, CB_1_R activation inhibits NMDAR signaling^28^, suggesting a comparable mechanism behind the repetitive stereotyped circling as NMDAR antagonists. However, another pivotal target of THC relating to its rewarding effects is the brain’s dopaminergic system^29^. Although the exact signal transduction path remains unknown, THC increases dopamine (DA) signaling along the mesolimbic pathway from the midbrain ventral tegmental area to the nucleus accumbens of the forebrain^29^. In addition to the above-mentioned glutamate hypothesis of schizophrenia, there is also long-standing empirical support for hyperactive DA signaling as a basis for psychosis etiology^23^.

Given the high prevalence of *Cannabis* use^1^ and its influence on both glutamatergic and dopaminergic neurotransmission, animal models of its psychotomimetic effects are valuable tools for elucidating the endocannabinoid system’s (eCBS) role in psychosis^30^. Zebrafish have a highly conserved eCBS and display neurobehavioral similarities with rodents following NMDAR antagonism^30,31^. As zebrafish lack DA neuronal expression in the midbrain, DA neurons in the basal diencephalon are a proposed functional counterpart to the mammalian mesolimbic DA system^32^. The considerable homology between the zebrafish CNS and the human CNS, combined with their rapid development, accessibility to molecular genetic dissection and *in vivo* imaging, make them an attractive choice in the biomedical field as they permit high-throughput screenings of genetic and pharmacological manipulations of embryos, larvae and adults^33–35^.

Making use of these beneficial traits, this present study firstly aims to produce and quantify THC’s effect on zebrafish stereotyped behavior using a newly developed computational method to quantify the Repetition Index (RI). Secondly, the effect of neurotransmitter imbalance on the behavioral stereotypy was investigated through co-administrations of THC with NMDAR agonist NMDA and GABA_A_ receptor antagonist pentylenetetrazol (PTZ) respectively. Thirdly, to validate if the behavioral stereotypy is mediated via CB_1_R or CB_2_R, THC was tested with the selective CB_1_R inverse agonist AM251 and with the selective CB_2_R inverse agonist AM630. Finally, to determine if the circular swimming is indicative of a psychotic state, THC was co-administered with the antipsychotic sulpiride. Overall, a zebrafish model of THC-induced behavioral stereotypies is presented, which is a valuable tool for future in-depth studies of the mechanistic relationship between *Cannabis* use and risk of mental illness at cellular and molecular levels.

## Results

### Tetrahydrocannabinol (THC) induces repetitive swimming patterns in adult zebrafish

**Figure 1:**
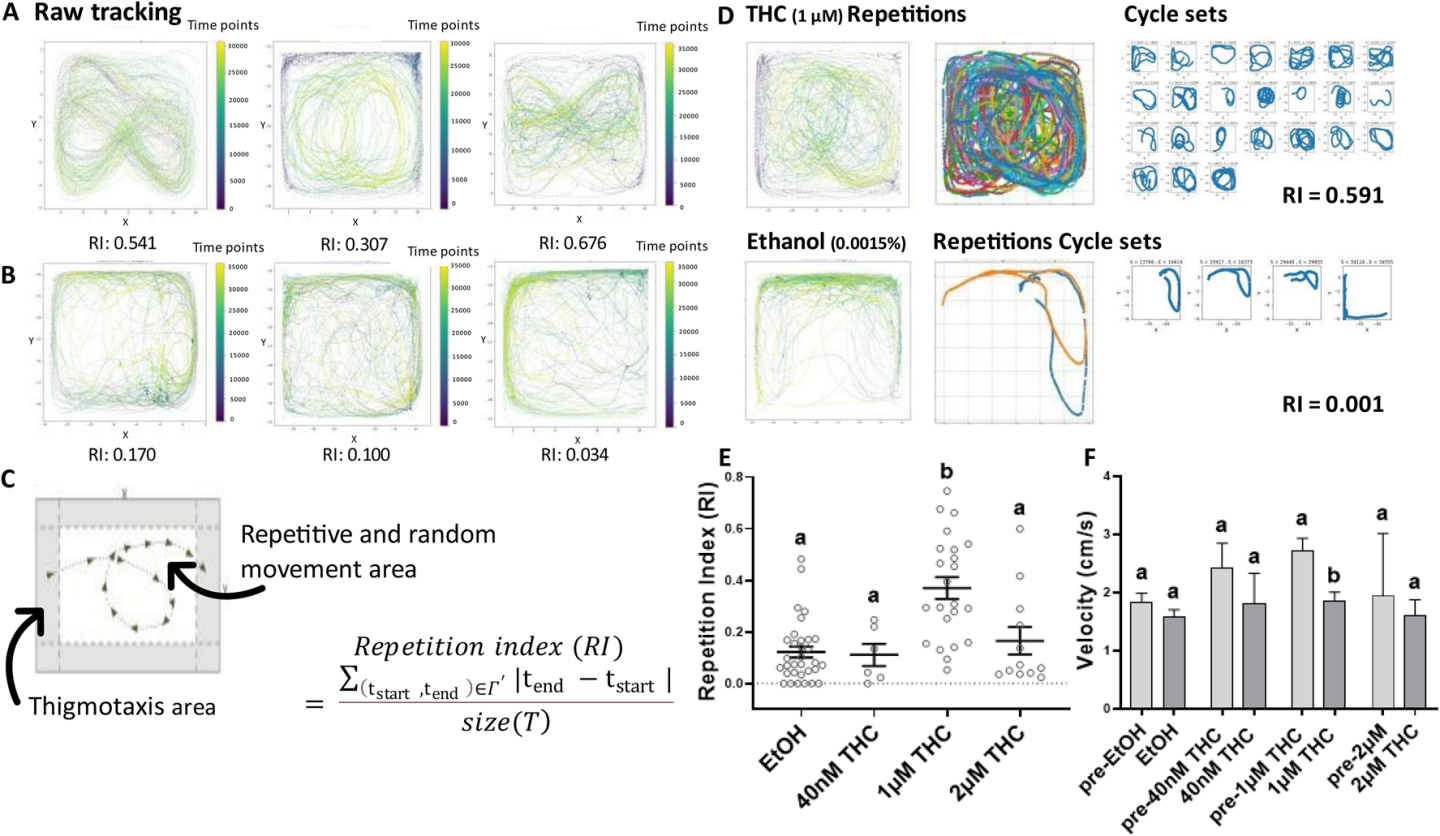
(**A**) Establishment of a Repetition Index (RI) uncovers quantifiable Tetrahydrocannabinol (THC)-induced behavioral stereotypy in adult zebrafish that differs from swimming patterns in the low concentration ethanol vehicle (**B**). (**C**) A schematic trajectory with inner and outer zones and formula to calculate the RI. (**D**) Repetitive patterns are extracted as cycle sets from the raw tracking and summed to give a RI value. (**E**) THC (1 μM) elicited a significantly greater RI than the control condition with EtOH (0.0015%) (****p < 0.0001), calculated as a mean ± SEM, (**F**) and caused a significant reduction in velocity during exposure (**p = 0.0077). Controls EtOH (n = 38), 40 nM THC (n = 6), 1 μM THC (n = 23) and 2 μM THC (n = 12). Time point *t* and the whole recording time interval *T.* Values without a letter in common are statistically different to the control condition (*p* < 0.05, Kruskal–Wallis and Dunn's multiple comparisons test).

**Figure 2:**
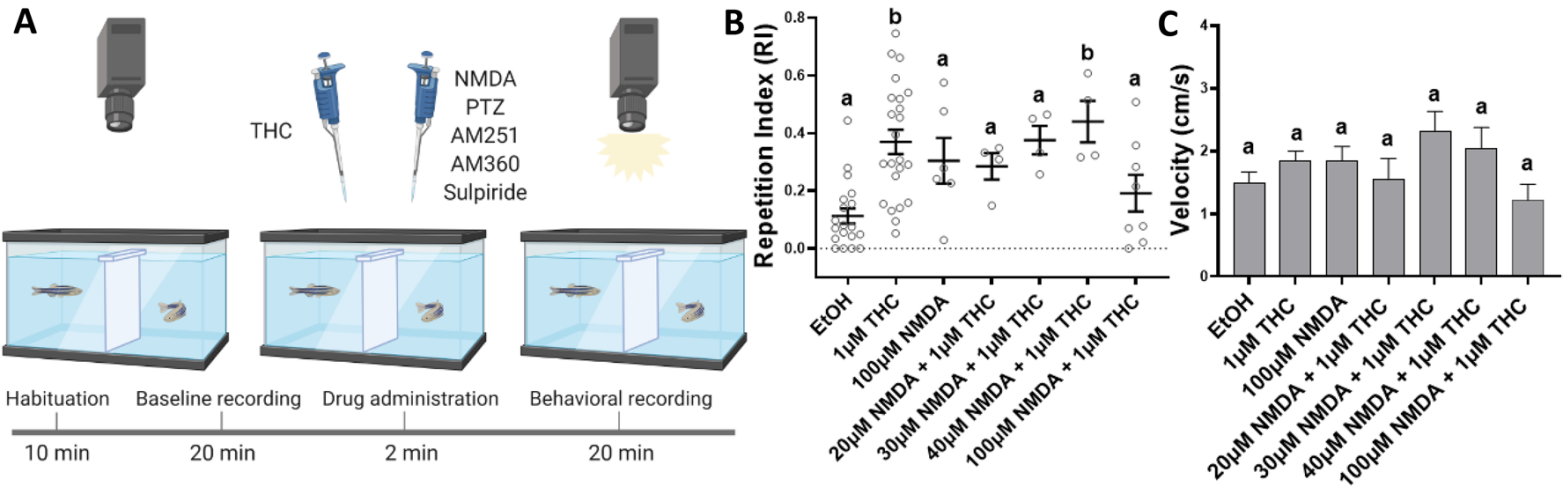
(**A**) Co-administration of Tetrahydrocannabinol (THC) and N-methyl-D-aspartate (NMDA) affects the THC-induced behavioral stereotypy in adult zebrafish. (**B**) At 100 μM NMDA with 1 μM THC the THC-induced Repetition Index (RI), calculated as a mean ± SEM, was not statistically different from the controls (EtOH) (ns *p* > 0.9999). (**C**) Nor did NMDA and THC co-administration affect the swimming velocity during exposure. Controls EtOH (0.0015%) (n = 19), 1 μM THC (n = 23), 100 μM NMDA (n = 6), 20 μM NMDA + 1 μM THC (n = 4), 30 μM NMDA + 1 μM THC (n = 4), 40 μM NMDA + 1 μM THC (n = 4) and 100 μM NMDA + 1 μM THC (n = 8). Mean RI values of 20 mM NMDA (n = 4), 30 mM NMDA (n = 4) and 40 mM NMDA (n = 4) were similar to 100 μM NMDA. Values without a letter in common are statistically different to the control condition (*p* < 0.05, Kruskal–Wallis and Dunn's multiple comparisons). Figure 2A was created with BioRender.com.

To determine the behavioral effects of THC, adult EK-WT zebrafish were individually immersed in 40 nM, 1 μM or 2 μM THC for 20 min and compared to control zebrafish exposed to the ethanol vehicle (0.00006%, 0.0015%, 0.003%) (Fig. 2A). No significant difference was found between the ethanol control concentrations (Supplementary Fig. S1) and therefore they were grouped together in Fig. 1E,F. However, in THC-treated individuals, we noted an abnormal behavioral pattern that had the characteristic of repetitive circling (Fig. 1A).

In order to measure this behavioral abnormality, we developed a computational method to quantify the repetition index (RI) (Fig. 1C and see “Methods”). The mean RI, the standard error of the mean (SEM) and the range for the control condition and the THC conditions were plotted in Fig. 1E. No difference was observed between males and females. THC evoked prominent circling behavior in 69.6% (n = 23) of fish at 1 μM, with a RI significantly higher than the ethanol controls (****p < 0.0001, Kruskal-Wallis; ****p < 0.0001, Dunn's multiple comparisons test) (Fig. 1E). The tracks of one fish at 40 nM THC were categorized as circular swimming but the mean RI was not significantly different from the controls (ns *p* > 0.9999, Dunn's multiple comparisons test). 2 μM THC elicited strong circling in 25% (n = 12) of the fish, but this was not sufficient to cause a significantly higher mean RI compared to the controls (ns *p* > 0.9999, Dunn's multiple comparisons test) (Supplementary Fig. S2).

There was no significant difference in mean velocity before or during exposure in the ethanol control, 40 nM THC or 2 μM THC conditions. At the concentration with the highest mean RI, 1 μM THC, velocity was significantly reduced during drug immersion (**p = 0.0019, Kruskal-Wallis; **p = 0.0077, Dunn's multiple comparisons test) (Fig. 1F). Taken together, 1 μM THC evoked the strongest circling behavior while simultaneously dampening overall velocity. Such dampening of velocity may be related to or independent of the circling behavior. The results from 1 μM THC administration in EK-WT fish (n = 23) and corresponding ethanol (0.0015%) controls (n = 19) were used in the subsequent experiments. Data from 11 fish were excluded due to failed tracking, leaving data from 73 fish.

### N-methyl-D-aspartate (NMDA) attenuates THC-induced behavioral stereotypy

Based on the results from the THC dosage tests (Fig. 1E,F), 1 μM THC was selected for the following experiments to examine if agonism of the NMDAR by NMDA could attenuate the THC behavioral stereotypy. 1 μM THC was co-administered with 20 μM, 30 μM, 40 μM, and 100 μM NMDA and compared to controls with ethanol (0.0015%) or NMDA at 20 μM, 30 μM, 40 μM or 100 μM. The mean RI, the SEM and the range for the control and experimental conditions were plotted in Fig. 2B. The highest concentration of NMDA alone (100 μM) was displayed in Fig. 2. The mean RI values for the lower concentrations of NMDA were: 20 μM NMDA, 0.561 (SEM 0.08, range: 0.454 to 0.797); 30 μM NMDA, 0.081 (SEM 0.06, range: 0 to 0.245); 40 μM NMDA, 0.304 (SEM 0.05, range: 0.233 to 0.449).

Figure 2B illustrates how 20, 30 and 100 μM NMDA given with 1 μM THC, diminished the mean RI to a value not significantly different from the controls (***p = 0.0003, Kruskal-Wallis; ns *p* > 0.05, Dunn's multiple comparisons test). However, fish exposed to 40 μM NMDA with 1 μM THC still exhibited a significantly higher mean RI (**p = 0.0068, Dunn's multiple comparisons test). At 100 μM NMDA and 1 μM THC, the behavior stereotypy was observed in 25% (n = 8) of the fish in comparison to 69.6% of fish at 1 μM THC alone. Moreover, none of the co-administrations of NMDA with 1 μM THC produced a significant change in velocity in comparison to the ethanol controls (ns *p* = 0.0581, Kruskal-Wallis) (Fig. 2C). Data from 8 fish were excluded due to failed tracking, leaving data from 80 fish.

### The GABA antagonist PTZ attenuates THC-induced behavioral stereotypy

**Figure 3:**
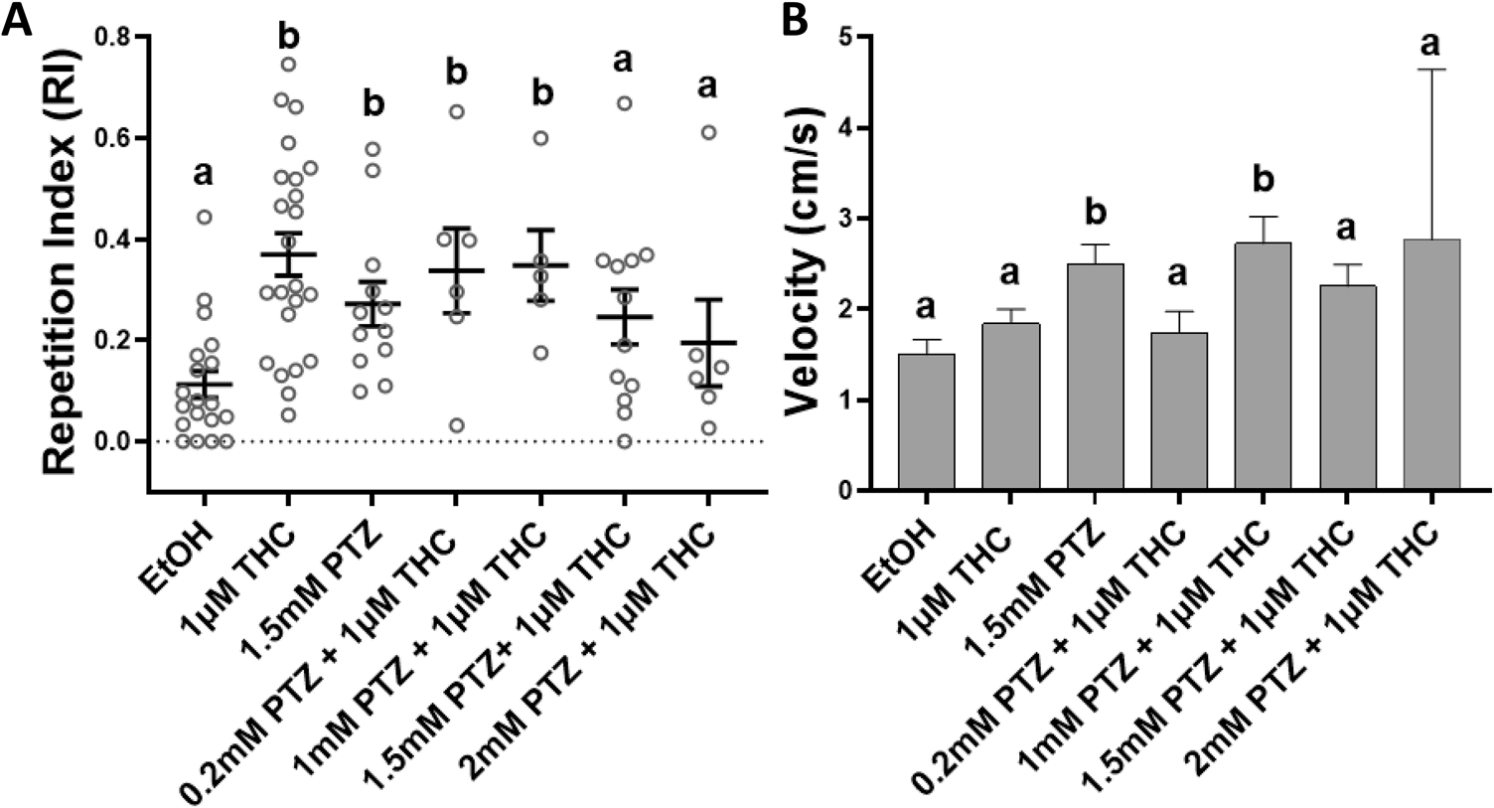
Co-administration of Tetrahydrocannabinol (THC) and GABA_A_ receptor antagonist pentylenetetrazol (PTZ) affects the THC-induced behavioral stereotypy in adult zebrafish. (**A**) At 1.5 mM PTZ with 1 μM THC the THC-induced Repetition Index (RI), calculated as a mean ± SEM, was not statistically different from the controls (EtOH) (ns *p* = 0.1578). (**B**) 1.5 mM PTZ and 1 mM PTZ with 1 μM THC caused significant increases in swimming velocity compared to controls (EtOH) (*p < 0.05). Controls EtOH (0.0015%) (n = 19), 1 μM THC (n = 23), 1.5 mM PTZ (n = 12), 0.2 mM PTZ + 1 μM THC (n = 6), 1 mM PTZ + 1 μM THC (n = 5), 1.5 mM PTZ + 1 μM THC (n = 12) and 2 mM PTZ + 1 μM THC (n = 6). Mean RI values of 0.2 mM PTZ (n = 6) and 2 mM PTZ (n = 6), were similar to 1.5 mM PTZ. Values without a letter in common are statistically different to the control condition (*p* < 0.05, Kruskal–Wallis and Dunn's multiple comparisons test).

Given that 100 μM NMDA showed signs of counteracting THC-induced repetitive circling behavior, PTZ was next co-administered with THC to measure the effect of reduced GABA transmission through GABA_A_R inhibition. The mean RI, the SEM and the range for the control and experimental conditions were plotted in Fig. 3A. The mean RI at 1 mM PTZ with 1 μM THC was 0.348 (SEM 0.07), whereas the RI was reduced to 0.246 (SEM 0.05), at 1.5 mM PTZ with 1 μM THC (Fig. 3A). At 2 mM PTZ, both alone and with 1 μM THC, the fish exhibited rapid swimming in a zig-zag pattern and convulsions indicative of PTZ’s seizure inducing effects (Supplementary Fig. S3)^36^. 1.5 mM PTZ alone was plotted in Fig. 3 as no convulsions were observed at this concentration.

Although the number of fish with a visible phenotype in the tracking was reduced from 69.6% in 1 μM THC alone to 50% (n = 6) and 40% (n = 5) with 0.2 mM and 1 mM PTZ respectively, the RI was still significantly higher than the controls (***p = 0.0005, Kruskal-Wallis; *p < 0.05, Dunn's multiple comparisons test). The discrepancy between the visual scoring and the RI is likely due to the RI method considering both the mean and duration of repetition. However, 1.5 mM PTZ with 1 μM THC was successful in restricting the stereotypy to 25% (n = 12) of the fish and generated a RI not statistically significantly different from the controls (ns *p* = 0.1578, Dunn's multiple comparisons test) (Fig. 3A). Nevertheless, the reduction in clear THC-circling with increasing PTZ doses may reflect the potent pro-convulsant effects of PTZ rather than a direct counteraction of THC’s effects. In comparison to the ethanol control condition, 1.5 mM PTZ alone and 1 mM PTZ with 1 μM THC caused noticeable increases in velocity (**p = 0.0071, Kruskal-Wallis; EtOH vs. 1.5 mM PTZ, **p = 0.0074; EtOH vs. 1mM PTZ + 1μM THC, *p = 0.0176, Dunn's multiple comparisons test) (Fig. 3B). Data from 7 fish were excluded due to failed tracking, leaving data from 83 fish.

### The selective CB_1_R inverse agonist AM251 does not significantly reduce THC-induced behavioral stereotypy

**Figure 4:**
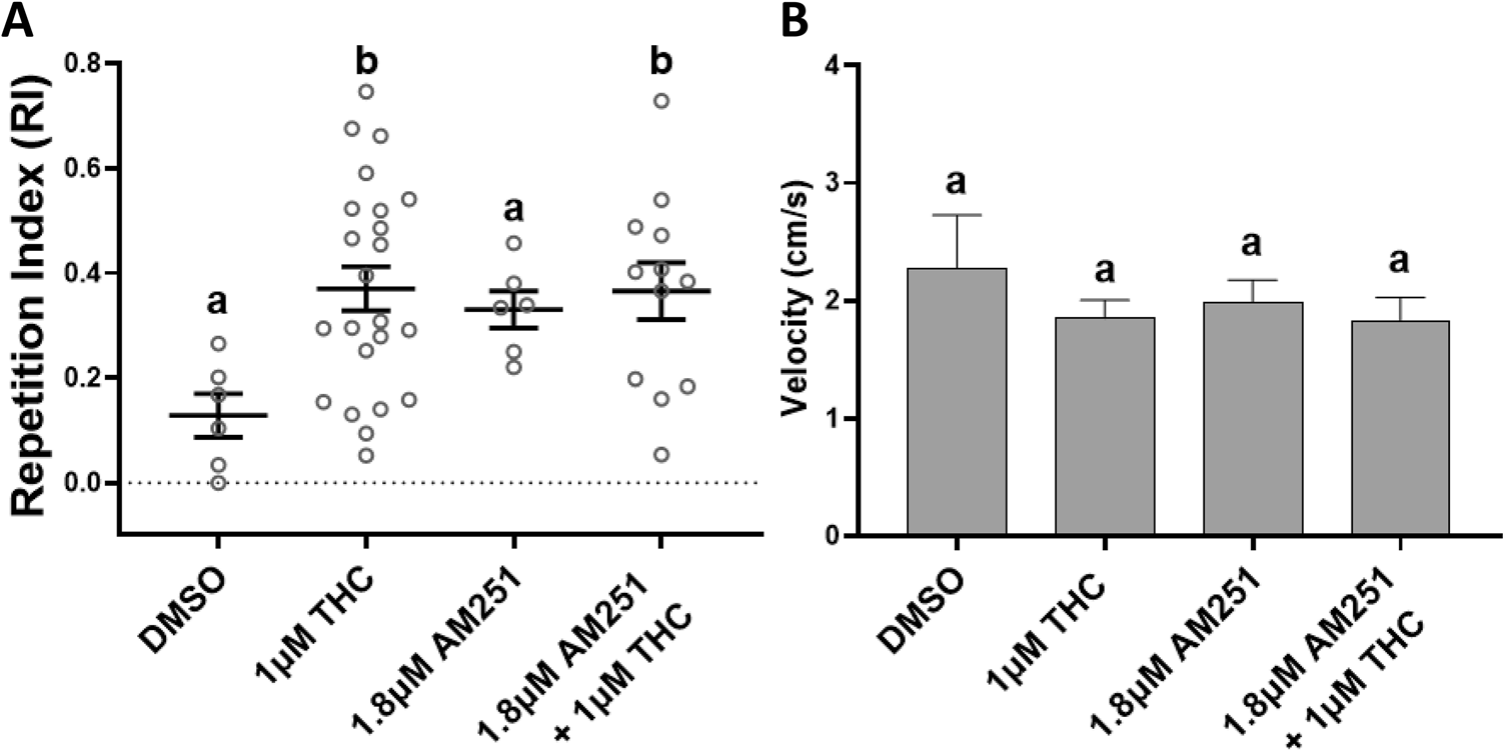
Co-administration of Tetrahydrocannabinol (THC) and the inverse CB_1_R agonist AM251 did not significantly reduce the THC-induced behavioral stereotypy in adult zebrafish. **(A**) At 1.8 μM AM251 with 1 μM THC the THC-induced Repetition Index (RI), calculated as a mean ± SEM, was statistically different from the controls (DMSO) (*p = 0.0279). (**B**) Nor did AM251 and THC co-administration affect the swimming velocity during exposure. Controls (1% DMSO, n = 6), 1 μM THC (n = 23), 1.8 μM AM251 (n = 6) and 1.8 μM AM251 + 1 μM THC (n = 12). Values without a letter in common are statistically different to the control condition (*p* < 0.05, One-way ANOVA and Dunnett's multiple comparisons tests for (A) and Kruskal–Wallis and Dunn's multiple comparisons test for (B)).

The selective CB_1_R inverse agonist AM251 at 1.8 μM was administered with THC to pharmacologically manipulate CB_1_R. The concentration of 1.8 μM AM251 was based on preliminary velocity tests (data not shown). The mean RI, the SEM and the range for the control and experimental conditions were plotted in Fig. 4A. At 1.8 μM AM251 with 1 μM THC the mean RI was significantly higher than the controls (DMSO) (*p = 0.0380, One-way ANOVA; *p = 0.0279, Dunnett's multiple comparisons test) and 58.3% (n = 12) of the fish exhibited stereotyped circling (Fig. 4A). 1.8 μM AM251 alone also elicited repetitive behavior detected by the algorithm but this was not visually analogous to the clear THC-circling (Fig. 1A). Neither the vehicle DMSO (1%), nor co-treatment with 1.8 μM AM251 significantly altered velocity (ns *p* = 0.5969, Kruskal-Wallis) (Fig. 4B). Data from 7 fish was excluded due to failed tracking, leaving data from 47 fish.

### The selective CB_2_R inverse agonist AM630 significantly attenuates THC-induced behavioral stereotypy

**Figure 5:**
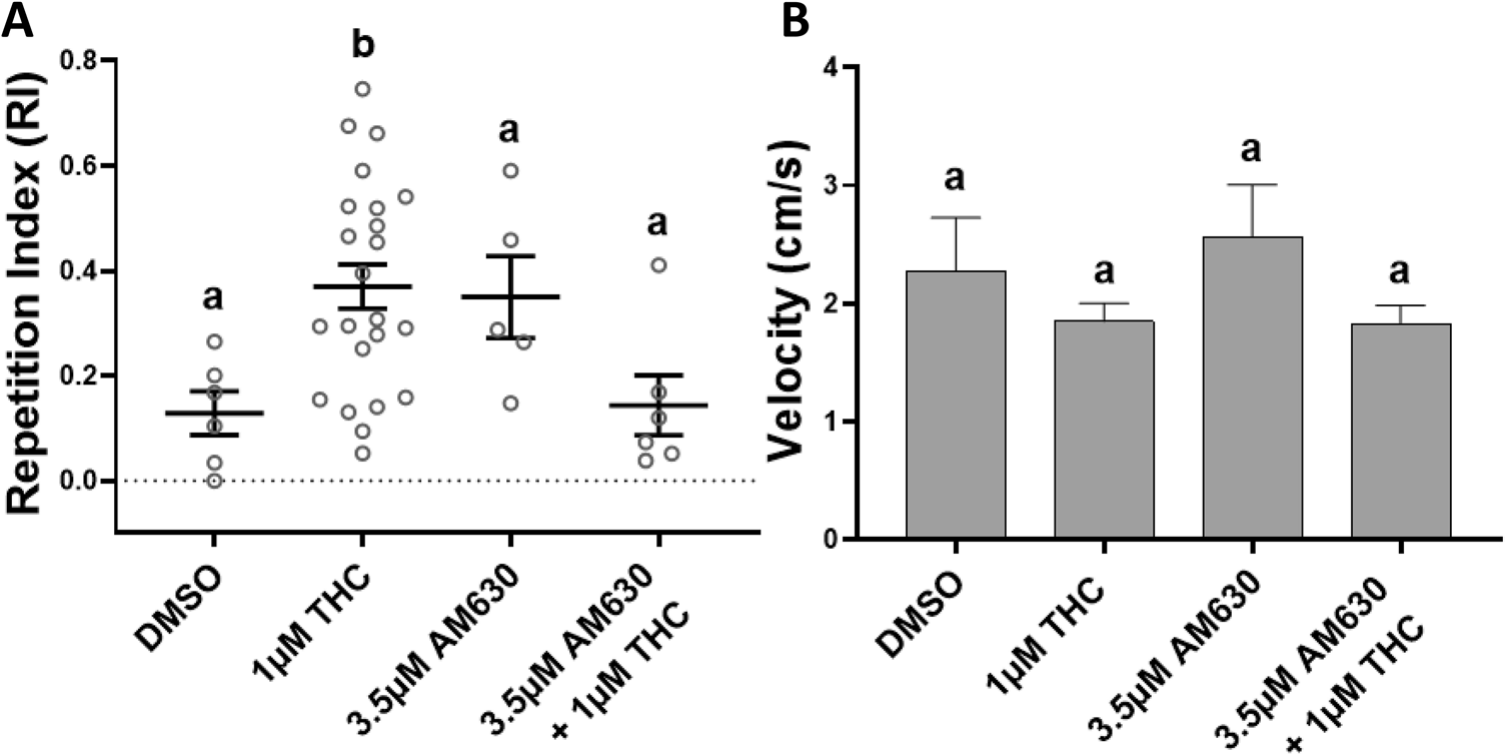
Co-administration of Tetrahydrocannabinol (THC) and the inverse CB_2_R agonist AM630 significantly reduced the THC-induced behavioral stereotypy in adult zebrafish. (**A**) At 3.5 μM AM630 with 1 μM THC the THC-induced Repetition Index (RI), calculated as a mean ± SEM, was not statistically different from the controls (DMSO) (ns *p* > 0.9999). (**B**) AM630 and THC co-administration did not affect the swimming velocity during exposure. Controls (DMSO 1%, n = 6), 1 μM THC (n = 23), 3.5 μM AM630 (n = 5) and 3.5 μM AM630 + 1 μM THC (n = 6). Values without a letter in common are statistically different to the control condition (*p* < 0.05, Kruskal–Wallis and Dunn's multiple comparisons test).

The negative indications regarding the role of CB_1_R in the behavioral stereotypy (Fig. 4), shifted the focus to CB_2_R and the co-treatment of THC with AM630, a selective CB_2_R inverse agonist. The experimental dose of 3.5 μM AM630 was selected based on previous work^37^ and DMSO (1%) control data from the AM251 testing was included in Fig. 5. With a mean RI of 0.144 (SEM 0.06), 3.5 μM AM630 with 1 μM THC reduced the repetitive circling to a level not statistically different from the controls, where 33.3% (n = 6) of the fish engaged in the behavior (**p = 0.0076, Kruskal-Wallis; ns *p* > 0.9999, Dunn's multiple comparisons test) (Fig. 5A). This was also found to be significantly lower than the mean RI of 1 μM THC alone (*p = 0.0296, Dunn's multiple comparisons test). 3.5 μM AM630 alone exhibited a relatively elevated mean RI of 0.350 (SEM 0.08), but there was no significant change in velocity when administered alone or with 1 μM THC (ns *p* = 0.3371, Kruskal-Wallis) (Fig. 5B). Data from 10 fish was excluded due to failed tracking, leaving data from 40 fish.

### The atypical antipsychotic sulpiride significantly attenuates THC-induced behavioral stereotypy

**Figure 6:**
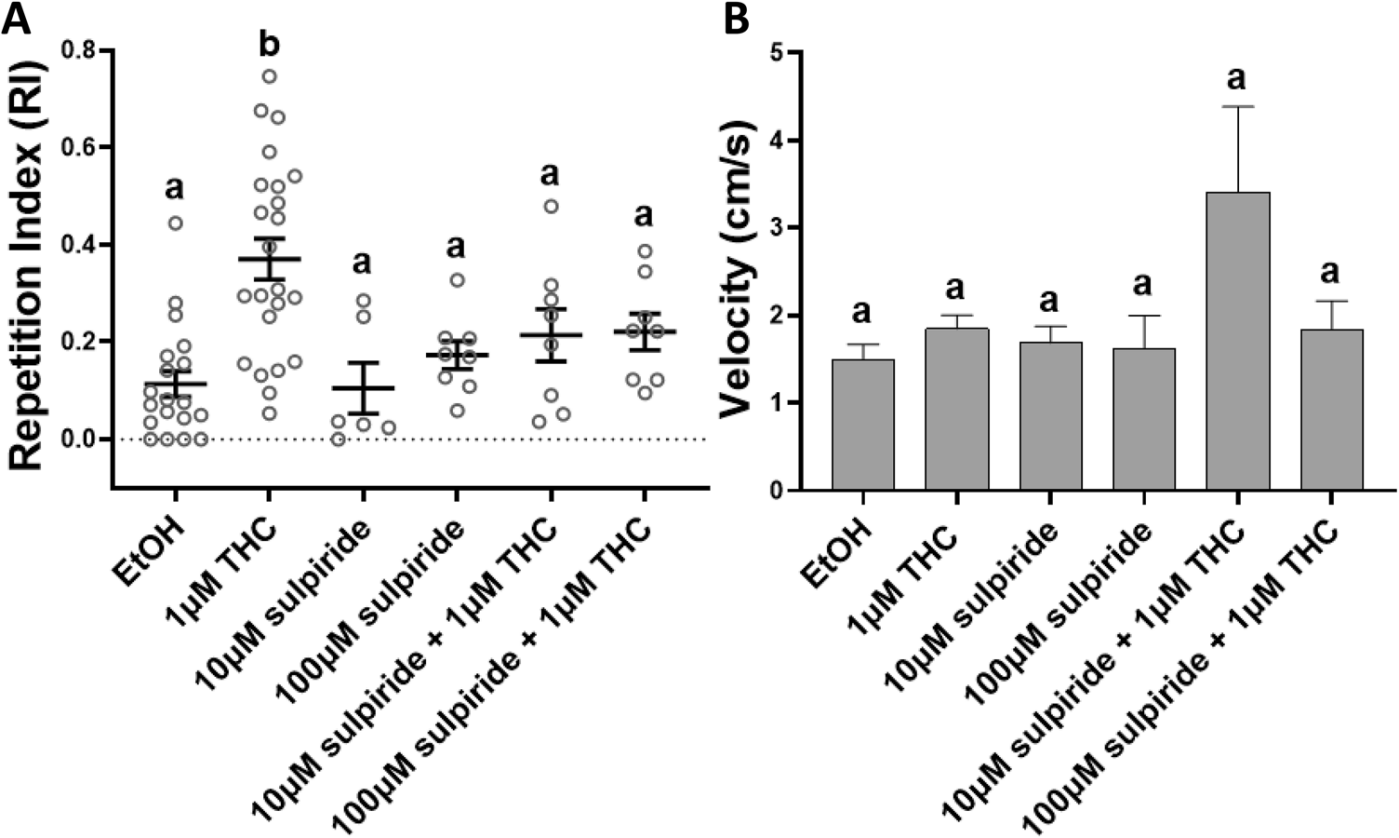
Co-administration of Tetrahydrocannabinol (THC) and the atypical antipsychotic sulpiride significantly reduced the THC-induced behavioral stereotypy in adult zebrafish. (**A**) At 10 μM sulpiride with 1 μM THC and 100 μM sulpiride with 1 μM THC, the THC-induced Repetition Index (RI), calculated as a mean ± SEM, was not statistically different from the controls (EtOH) (ns *p* > 0.05). (**B**) None of the co-administrations significantly altered the swimming velocity during exposure (ns *p* > 0.05). Controls (EtOH) (0.0015% (n = 19)), 1 μM THC (n = 23), 10 μM sulpiride (n = 6), 100 μM sulpiride (n = 8) 10 μM sulpiride + 1 μM THC (n = 8) and 100 μM sulpiride + 1 μM THC (n = 8). Values without a letter in common are statistically different to the control condition (*p* < 0.05, Kruskal–Wallis and Dunn's multiple comparisons test).

To examine THC-induced circling as a psychosis-like phenotype, EK-WT fish were given 1 μM THC with the antipsychotic sulpiride. The mean RI, the SEM and the range for the control and experimental conditions were plotted in Fig. 6A. Both 10 μM and 100 μM sulpiride co-administered with 1 μM THC significantly lowered the repetitive circling (12.5% (n = 8) and 25% (n = 8) respectively), to a mean RI not significantly higher than the controls (***p = 0.0001, Kruskal-Wallis; ns *p* > 0.05, Dunn's multiple comparisons test). Both co-administrations of sulpiride and THC weakened the circling without significantly reducing the velocity of the fish during exposure (ns *p* > 0.05) (Fig. 6B). 10 μM and 100 μM sulpiride alone also did not influence locomotion. An increase in velocity was measured during application of 10 μM sulpiride with 1 μM THC, although this was not found to be statistically significant (ns *p* = 0.3434, Kruskal-Wallis; ns *p* = 0.2009, Dunn's multiple comparisons test) (Fig. 6B). Data from 8 fish was excluded due to failed tracking, leaving data from 72 fish.

## Discussion

Using a new analytical method that we have developed, this study demonstrated that 1 μM THC administration in adult zebrafish triggered a shift from typical navigational locomotor patterns to a repetitive circling behavior, which was ameliorated by the antipsychotic sulpiride (Figs. 1 and 6). This behavioral phenotype appears analogous to THC’s effect in rats^24^ and the effect of NMDAR antagonists in zebrafish models of psychosis^16,17,38^. Notably, it did not occur in the ethanol control group or in the experimental conditions without THC. Harnessing this behavioral stereotypy through a quantitative measure of RI rather than through manual scoring, eliminates issues of experimenter bias and broadens the possibilities of standardized screens of antipsychotic drugs and for clarifying the enigmatic relationship between endocannabinoids and psychosis/schizophrenia.

*Cannabis* has had a medicinal role for millennia^39^ and has lower dependence potential (8.9%) compared to other common drugs of abuse like nicotine (67.5%) or alcohol (22.7%)^40^. Theories connecting *Cannabis*-use and psychotic episodes began to surface in the 1980s and since then, research has put forward bidirectional associations between *Cannabis* consumption and psychosis, where high frequency use, early onset of use and use of *Cannabis* containing high THC concentrations (12-18%) act as mediating factors^9,41–43^. The susceptibility to psychosis-like symptoms varies across *Cannabis* consumers as it involves a complex interplay between environmental factors and genetic predispositions^44^. Polymorphisms of genes involved in DA metabolism, e.g. COMT and DAT1, are of reoccurring interest as they may increase the vulnerability to neuronal over-excitation by DA in the prefrontal cortex (PFC) and give rise to executive dysfunctions and psychoses^45–47^. As cannabinoids increase dopaminergic signaling, by interrupting glutamate and GABA neurotransmission, *Cannabis*-use may entail long-term risks in those with dysfunctional DA metabolism^44^.

*Cannabis* is an atypical drug with contradicting responses, especially in zebrafish where there are reports of anxiogenic effects in adults^48^ and biphasic responses in larvae^49^ depending on the dosage. Here we present a concentration-dependent THC-induced behavioral stereotypy which is partially attenuated by NMDA, in a non-linear fashion (Fig. 2A). This hints of an indirect glutamate modulation of the behavioral phenotype in question, corroborating previous zebrafish studies with the NMDAR antagonists PCP, ketamine and MK-801^16,17,50^. The pharmacological amplification of NMDAR excitation and thereby an increased glutamate release, may have counteracted THC’s NMDAR antagonism. Likewise, inhibiting GABA_A_R using PTZ showed trends of lowering the RI (Fig. 3A). A combined depression of glutamate by THC and GABA by PTZ could have maintained the excitation/inhibition balance of the CNS and prevented repetitive circular locomotion. However, the potent nature of PTZ caused convulsions at 2 mM (Supplementary Fig. S3). Therefore, RI reductions could be due to a general PTZ effect on locomotion and not a direct counteraction of THC (Fig. 3A)^36^. Expanding the dose response analysis of THC, NMDA and PTZ and performing absorption, distribution, metabolism and excretion (ADME) analysis in zebrafish will shed further light on the observed concentration-dependent effects.

Regardless of the possible THC-mediated shift in CNS excitation/inhibition balance, THC’s effect on the current behavioral phenotype appeared to be CB_1_R-independent and CB_2_R-dependent in zebrafish. The CB_1_R specific inverse agonist AM251 was ineffective at lowering the RI when co-administered with THC, to a value not significantly different from the control condition (Fig. 4A). This was surprising as it contradicts CB_1_R’s central role in cannabinoid modulation of rodent locomotion, cognition, behavior and reports of CB_1_R antagonists reversing THC’s effects^51,52^. CB_1_R is also known to directly regulate NMDAR via the HINT1 protein^53^, and is colocalized with cholecystokinin (CCK) basket cells, a type of GABA interneuron in the PFC^54^. Through these interactions, CB_1_R agonists may diminish NMDAR activity and inhibit GABA release from CCK-basket cells, leading to a disinhibition of excitatory pyramidal cells^55,56^. Consequently, downstream DA excitation is potentiated and causes an imbalance in cortical functioning, which is a clinical feature of schizophrenia^57^.

Despite the multitude of CB_1_R pathways for THC to exert its effects on glutamate, GABA and downstream DA signaling, reports of THC as a multitarget ligand may better explain the non-CB_1_R mediated THC behavioral stereotypy^58^. The CB_2_R inverse agonist AM630 given with 1 μM THC reduced the frequency of circling and significantly lowered the mean RI of 1 μM THC alone to a RI not significantly different from the controls (Fig. 5A). In addition, AM630 prevented the THC-related reduction in velocity during immersion (Fig. 5B). CB_2_R modulation of zebrafish locomotion is complex, as larvae lacking CB_2_R have been shown to swim less in light periods and more in dark^37^. The CB_2_R (-/-) knockouts (KOs) also avoided open spaces, thereby displaying an anxiety-like behavior compared to WT larvae^37^. Zebrafish carry two CB_2_R duplicates (cb2a and cb2b), as opposed to one CB_1_R, that could exhibit different functional activities compared to CB_2_R of other species^59^.

Although CB_2_R are mainly expressed in immune cells of the peripheral nervous system^26^, their expression has also been reported in the central nervous system, e.g., midbrain dopamine neurons^27^. Associations between the single nucleotide polymorphisms rs12744386 and rs2501432, which impair the function of the *CNR2* gene encoding CB_2_R, and an enhanced risk of schizophrenia have been reported^60^. Additionally, reduced reflex responses in the pre-pulse inhibition (PPI) test, where a subthreshold stimulus precedes a startle stimulus, have been established in both schizophrenic patients^61^ and in mice lacking CB_2_R^62^. The antipsychotic risperidone restores PPI in CB_2_R KOs which paints a possible role for CB_2_R in psychosis-like behaviors^62^. This warrants future experiments with adult zebrafish lacking CB_2_R and structurally dissimilar CB_2_R antagonists to further examine the CB_2_R’s potential action in the phenotype of interest and psychosis.

**Figure 7:**
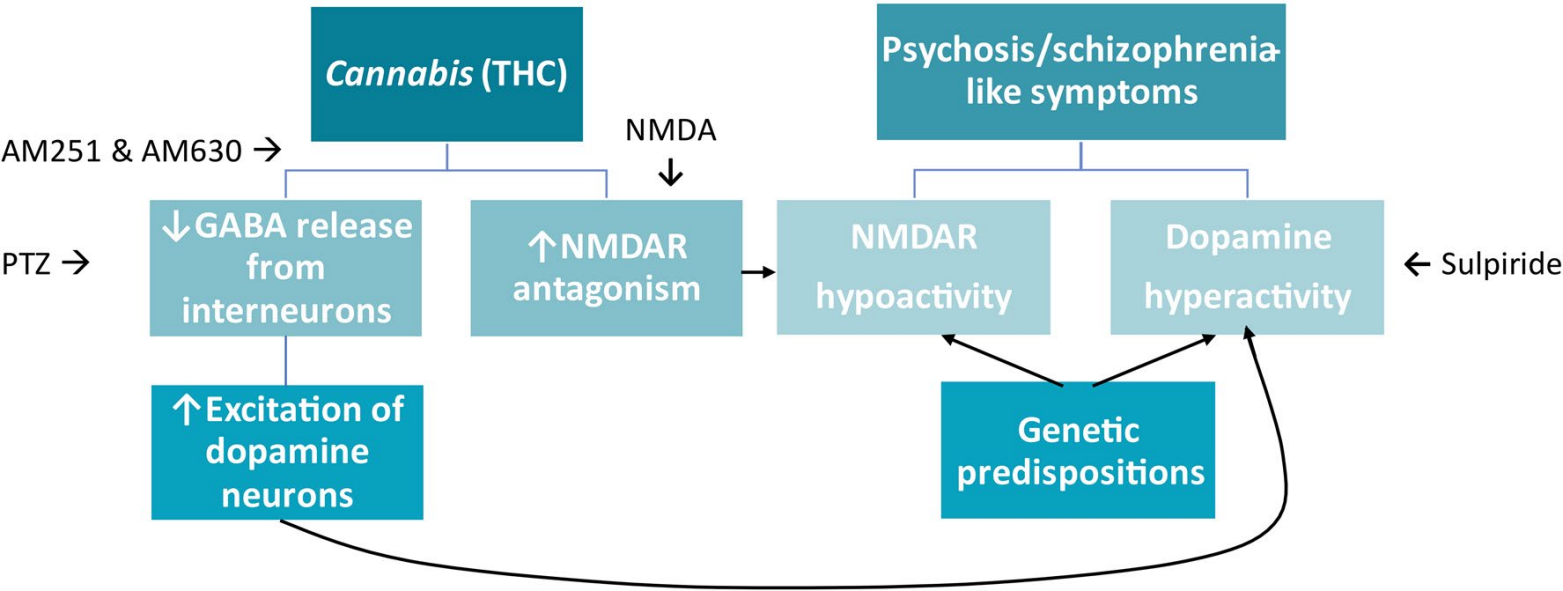
Potential mechanisms underlying the associations between *Cannabis* consumption and risks of psychosis/schizophrenia-like symptoms, with the current study’s pharmacological approaches. Δ9-tetrahydrocannabinol (THC) reduces N-methyl-D-aspartate (NMDA) release via NMDA receptor antagonism. THC also inhibits γ‐aminobutyric acid (GABA) release from interneurons in the prefrontal cortex, potentiating dopamine release. Both NMDA receptor hypoactivity and heightened dopaminergic signaling are hallmarks of schizophrenia. Pentylenetetrazol (PTZ).

Promising support for the circular swimming mimicking schizophrenia-like symptoms was obtained in the sulpiride tests (Fig. 6). Sulpiride is an atypical antipsychotic that inhibits central DA D_2_ receptors and acts to dampen the disorder’s DA hyperactivity^63^. Both 10 μM and 100 μM sulpiride with 1 μM THC lowered the mean RI of 1 μM THC alone to a RI not significantly different from the controls (Fig. 6A). Importantly, sulpiride alone and with THC did not significantly influence the overall velocity of the fish (Fig. 6B). Atypical antipsychotics have been successful in reversing additional aspects of schizophrenia-like behavior, such as cognitive impairment and social withdrawal, induced by NMDAR antagonist MK-801 in zebrafish (sulpiride)^63^ and rats (aripiprazole)^64^. One of the downstream effects of their serotonergic and dopaminergic antagonism is NMDAR activation via d-serine release in the PFC^65,66^. The polypharmacology of atypical antipsychotics may therefore explain their efficacy, by simultaneously targeting the DA hypothesis and the glutamate hypothesis of schizophrenia^63,65^. Similarly, THC’s discussed mechanisms of action are also intertwined with both hypotheses, making it difficult to pinpoint a direct cause-effect relationship (Fig. 7). Future co-treatments of THC with other atypical antipsychotics, such as clozapine, will further strengthen these notions^66^.

With any animal model of complex disorders and diseases there is always the question of face validity and construct validity, i.e., how well the model resembles and measures the illness^67^. One approach to address the complexity issues in gene-behavior interactions is to focus on endophenotypes, which concentrate on a specific heritable characteristic and its circuitry such as the PPI deficit in schizophrenia^68,69^. Future experiments to further strengthen the THC-induced behavioral stereotypy as an endophenotype of psychosis include tests in zebrafish lacking CB_2_R or carrying mutations linked to psychosis (e.g. *RBM12*)^70^ or addiction (e.g. *SLIT3*)^34^.

Another limitation of using a newly established analytical method is that it lacks validation across different data sets. Further optimization of our newly developed algorithm and machine learning would allow better detection and extraction of repetitive patterns and bridge the gap between distinct behavior detected by the human eye and patterns detected by the computer. Tailored RI measures for abnormal repetitive behaviors can greatly improve assays such as the current one and lay a foundation for an automated analysis with standardized behavioral endpoints^67^. This in turn can assist in further validating the behavioral stereotypy as an endophenotype for THC-induced psychosis. From there, the search for its genetic underpinnings and pharmacological interventions can be pursued.

In conclusion, zebrafish engage in intriguing concentration-dependent swimming patterns when immersed in THC, which share characteristics with other animal models of drug induced psychosis- and schizophrenia-like behaviors. NMDA showed signs of counteracting THC’s effect, and surprisingly this appeared to be CB_1_R-independent but CB_2_R-dependent. As sulpiride reduced the repetitive swimming, the THC-elicited behavior may indicate a psychosis-like state.

## Methods

### Animals and housing

For the adult THC dosage tests and the THC co-administration with NMDA, PTZ, AM251, AM630 and sulpiride, zebrafish of the EK-WT strain, a wild-type line obtained from Ekkwill Breeders in Florida, were used (aged 9-12 months, n = 258, 50:50 male to female ratio). Upon arrival, the EK-WT fish had a three-week habituation period in the facility at the University of California, San Francisco, USA. The fish (mean length = 3.55 cm, body weight = 0.87 g) were housed in groups of 5-7 fish per tank (26.5 cm (L) x 8 cm (W) x 17 cm (H), ca 2 L volume) separated by gender.

Adult fish were fed twice per day with flake food (Tropical Flakes, Aquatic Eco-Systems) and live brine shrimp (Platinum-Grade Argentemia Brine Shrimp, Argent Chemical Laboratories). The fish facility was kept at 28 °C with a 14hr/10hr light/dark cycle. The system water contained 5 g of Instant Ocean Salts (Aquatic Eco-systems) and 3 g sodium bicarbonate per 20 L of reverse-osmosis water (pH 7.6).

### Chemicals

Δ9-tetrahydrocannabinol (THC, schedule I controlled substance) was supplied by the National Institute on Drug Abuse and stored in vials of 20 mg/ml THC in 95% ethanol. N-Methyl-D-aspartic acid (NMDA, cat. no. M3262-100MG), Pentylenetetrazole (PTZ, cat. no. P6500-25G) and (±)-sulpiride (cat. no. S8010-25G) were purchased from Sigma-Aldrich and diluted in MilliQ water to the desired concentrations. AM251 (Abcam, cat. no. ab120088) and AM630 (Sigma-Aldrich, cat. no. SML0327-5MG) were diluted in dimethyl sulfoxide (DMSO, Sigma-Aldrich, cat. no. 317275-500ML). Ethanol (Rossville Gold Shield Ethyl Alcohol, cat. no. 94545) was purchased from Gold Shield Chemical.

### Behavioral recordings

Behavioral testing was carried out in a cabinet (Supplementary Fig. S4) constructed specifically for the study (materials purchased from McMaster-Carr Supply Company). A Styrofoam board lined the bottom of the set-up to insulate from noise and a dark curtain allowed consistent experimental lighting. Lights and two cameras (Panasonic) were mounted from the top of the cabinet. The cameras were connected to a PC with BlueIris 4 recording software (Perspective Software).

### THC dosage tests

Naïve EK-WT fish (n = 84, 42 females, 42 males) were singly housed 5 days prior to the experiment. On the day of the experiment the fish were placed in the testing room (27.1°C) to habituate for 1 hr. White noise was provided from a fan. Individual fish were gently netted, with minimal distance to prevent hypoxia, into white tanks containing 0.7 L system water. The testing tanks were divided into two compartments by a white partition (Supplementary Figs. S4C, S4D), allowing two fish of the same gender to be tested in the same tank. After a 10 min habituation period, the fish were recorded for 20 min to determine baseline locomotion behavior. Next, 7 ml of THC (4 μM, 0.1 mM, 0.2 mM) or ethanol (0.006%, 0.15%, 0.3%) was added to each tank from 100-fold more concentrated stock solutions made fresh daily. The final THC concentrations in the tanks were 40 nM (n = 6), 1 μM (n = 24) and 2 μM (n = 12). The final ethanol concentrations in the control tanks (0.00006% (n = 6), 0.0015% (n = 24) and 0.003% (n = 12)) corresponded to the ethanol concentrations in the THC conditions. Preliminary dose testing was done by group exposing fish to 40 nM, 200 nM, 1 μM, 2 μM and 5 μM THC. 20-min recordings were performed to determine locomotion behavior during THC-exposure. Following the 20-min recording during THC-exposure, fish were passed through system water to rise off any remaining drug and returned to their housing tanks. Movement of the fish was quantified using the video-tracking software Ethovision XT 13. All tests were performed between 9 am and 5 pm.

### THC co-administrations with NMDA, PTZ, AM251, AM630 and sulpiride

Naïve EK-WT fish (n = 174, 87 females, 87 males) were individualized, habituated and tested in the same manner as the THC-dosage testing. After a 20-min recording of baseline locomotion behavior, THC was co-administered with NMDA, PTZ, AM251, AM630 and sulpiride respectively at 100-fold more concentrated stock solutions made fresh daily. The final NMDA concentrations in the testing tanks were 1 μM THC with 20 μM (n = 4), 30 μM (n = 4), 40 μM (n = 4) and 100 μM NMDA (n = 8). Control fish were exposed to NMDA alone at the same concentrations with the same sample size (n = 24). PTZ concentrations in the testing tanks were 1 μM THC with 0.2 mM (n = 6), 1 mM (n = 6), 1.5 mM (n = 12) and 2 mM PTZ (n = 6). Control fish were exposed to PTZ alone at the same concentrations, except 1 mM PTZ, with the same sample size (n = 24). The concentration of 1.8 μM AM251, diluted in DMSO (≥99%), with 1 μM THC (n = 12), was based on preliminary experiments. 1.8 μM AM251 alone (n = 6) and DMSO (1%) alone (n = 12) served as controls. 3.5 μM AM630 was also diluted in DMSO (≥99%) and given alone (n = 6) and with 1 μM THC (n = 8). For sulpiride, the final concentrations were 1 μM THC with 10 μM (n = 8) and 100 μM sulpiride (n = 8). The controls were given 10 μM (n = 8) and 100 μM sulpiride (n = 8) alone. After 20-min recordings of drug exposure, fish were rinsed with system water and returned to their housing tanks. Tests were performed between 9 am and 5 pm and water containing drugs was disposed of in accordance with Drug Enforcement Administration guidelines.

### Analysis, calculations, graphs and statistics

The behavioral recordings were analyzed by Ethovision XT 13 using the swim velocity parameter. Graphs were plotted using GraphPad Prism 9.1, experimental flow chart (Fig. 2A) was created using BioRender.com, and Fig. 7 using Microsoft PowerPoint. Normality of data sets was tested using the Shapiro-Wilk test. For normally distributed data sets, one-way ANOVAs and Dunnett's multiple comparisons tests were used. For non-parametric data, Kruskal-Wallis and Dunn’s multiple comparisons tests were applied. *p*-values less than 0.05 indicate significance.

The raw x and y co-ordinates from the inner zone (Fig. 1C) of the Ethovision tracking were used to calculate an unbiased Repetition Index (RI) using the following algorithm in Python and Spyder:

Initialization: Select the animal movement trajectory $M$, where $M = \left( {x\left( t \right),y\left( t \right)} \right), t \in T$ and $t$ is a time point and $T$ is the whole recording time interval.

Set the temporal and spatial threshold values as follows:

- $\theta_{time - window}$: sliding time window ($\theta_{time - window} =$ 500 time points),
- $\theta_{repetition - time}$: repetition time intervals ($\theta_{repetition - time} =$ 700 time points),
- $\theta_{thigmotaxis - margin}$: percentage of the movement area dedicated to the thigmotaxis margin ($\theta_{thigmotaxis - margin} = 0.1)$,
- $\theta_{std - change}$: minimum change in standard deviation to detect the repetitive movements ($\theta_{std - change} =$ 0.01).

1. Detect the non-thigmotaxis time intervals:
  $$ \begin{aligned} & x^{\prime } \left( t \right) = \{ x\left( t \right) if x\left( t \right) > \max \left( x \right) - \theta_{thigmotaxis - margin} \left( {\max \left( x \right) - \min \left( x \right)} \right)\} \\ & x^{\prime } \left( t \right) = \{ x\left( t \right) if x\left( t \right) < \min \left( x \right) + \theta_{thigmotaxis - margin} \left( {\max \left( x \right) - \min \left( x \right)} \right)\} \\ & y^{\prime } \left( t \right) = \{ y\left( t \right) if y\left( t \right) > \max \left( y \right) - \theta_{thigmotaxis - margin} \left( {\max \left( y \right) - \min \left( y \right)} \right)\} \\ & y^{\prime } \left( t \right) = \{ y\left( t \right) if y\left( t \right) < \min \left( y \right) + \theta_{thigmotaxis - margin} \left( {\max \left( y \right) - \min \left( y \right)} \right)\} \\ \end{aligned} $$
2. Calculate the standard deviation of x and y in all sliding window intervals:
  $$ \begin{array}{*{20}c} {std_{{x^{\prime } }}^{t} = std_{{x^{\prime } }} \left( {t:t + \theta_{time - window} } \right),} \\ {std_{{y^{\prime } }}^{t} = std_{{y^{\prime } }} \left( {t:t + \theta_{time - window} } \right),} \\ {t \in T} \\ \end{array} $$
3. Repetitive movement interval detection:
  $$ Rep\left( t \right) = \left\{ {\begin{array}{*{20}c} {1\; if\; \left| {std_{{x^{\prime } }}^{t} - std_{{x^{\prime } }}^{t + 1} } \right| < \theta_{std - change} , } \\ {0 \quad else,} \\ {t \in T } \\ \end{array} } \right. $$
4. Calculate the repetitive time intervals:
  $$ {\Gamma } = \left\{ {\left( {{\text{t}}_{{{\text{start}}}} ,{\text{t}}_{{{\text{end}}}} } \right)\;{\text{where}}\;Rep\left( i \right) = = 1\;{\text{if}}\;{\text{t}}_{{{\text{start}}}} \le {\text{i}} \le {\text{t}}_{{{\text{end}}}} { }} \right\} $$
5. Remove the random movements (obtain the repetitive cycles):
  $$ \Gamma^{\prime } = \{ \left( {{\text{t}}_{{{\text{start}}}} ,{\text{t}}_{{{\text{end}}}} } \right)\;\; \in {\Gamma }\;{\text{if}}\;{ }|{\text{t}}_{{\text{e}}} - {\text{t}}_{{\text{s}}} | > \theta_{repetition - time} \} $$
6. Calculate the Repetition index (RI):
  $$ RI = \frac{{\mathop \sum \nolimits_{{\left( {{\text{t}}_{{{\text{start}}}} ,{\text{t}}_{{{\text{end}}}} } \right) \in \Gamma^{\prime } }} |{\text{t}}_{{{\text{end}}}} - {\text{t}}_{{{\text{start}}}} |}}{size\left( T \right)} $$

Optimization: To detect and extract repetitions in the movement trajectory *M* (Fig. 1), the algorithm was optimized with a sliding window size of 500 time points (θ_time-window_) (2:45 min), a minimum repetitive behavior threshold of standard deviation = 0.01 (<θ_std-change_), and a repetitive interval threshold of 700 time points (θ_repetition-time_) (3:51 min). The thresholds were determined by trialing different values and a) visually comparing how well the extracted cycle sets (e.g, Fig. 1D) captured the repetitive movements and b) how well the RI value reflected the repetitive behavior (i.e., a higher value for strong circling and a lower value for random swimming trajectories). Anonymized tracking images (e.g., Fig. 1A,B) were independently sorted into high and low repetitive behavior by two researchers. If discrepancies between the manual sorting and the corresponding RI values occurred, the algorithm’s threshold values were adjusted.

(1) 10% of the total distance across the x and y axes, near the edges of the tank, were designated as thigmotaxis margins (Fig. 1C). Detected movement in this region was removed. (2) The standard deviation between the x and y co-ordinates within the specified window was calculated (3) and if below 0.01 (<θ_std-change_), the trajectory was considered repetitive and set to 1, i.e. the fish returns to the same co-ordinates during the time frame. If the standard deviation was above the threshold, the trajectory was considered arbitrary and set to 0. (4) Next, if the event set to 1 had a duration longer than 700 time points (>θ_repetition-time_), it was extracted as a cycle set (Fig. 1D), (5) while shorter events < θ_repetition-time_, were excluded (Supplementary Fig. S5). Thus, the standard deviation between the x and y co-ordinates within the specified window had to be close to 0 and the minimum duration of the behavior 3:51 min in order for the algorithm to extract the behavior. (6) The durations of all cycle sets were summed, divided by the total time interval *T* and normalized into RI values ranging between 0 to 1. Higher RI values signify intensified and prolonged repetitions in swimming trajectory, such as the circling or eight-shaped patterns (Fig. 1A), whereas values nearer 0 indicate more random movement (Fig. 1B).

After each experiment the tracking images, such as Fig. 1A,B, were anonymized and randomized to allow for manual selection of prominent circling behavior. This observational data is given as a percentage of fish with distinguished circling within the experimental cohort.

### Ethical confirmation statements

All husbandry and experimental methods were carried out in accordance with relevant guidelines and regulations: National Institutes of Health’s (NIH) principles for the care and use of animals in experimental procedures. All experimental protocols were approved by the Institutional Animal Care and Use Committee of the University of California, San Francisco. The experimental design and its description here, adhered to the ARRIVE guidelines^71^ for reporting animal research.

## Supplementary Information

Supplementary Information.

## Data Availability

Code for data analysis is provided at https://github.com/Mahdizarei/Repetitive-behavior.
