# Supplementary material for: THC-induced behavioral stereotypy in zebrafish as a model of psychosis-like behavior
Source: Sci Rep. 2021 Aug 3;11:15693. doi: 10.1038/s41598-021-95016-4 (PMC8333334; doi:10.1038/s41598-021-95016-4)
Supplement: Supplementary file 1 — Supplementary Information. [file 41598_2021_95016_MOESM1_ESM.pdf]

THC-induced Behavioral Stereotypy in Zebrafish as a Model of Psychosis-like Behavior

Amelia Dahlén, Mahdi Zarei, Adam Melgoza, Mahendra Wagle, and Su Guo

Supplementary information:

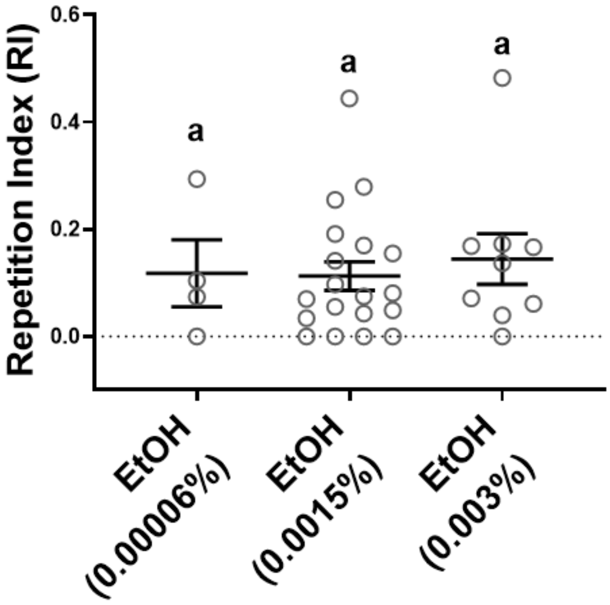

Figure S1. The effect of ethanol vehicle (0.00006% - 0.003%) on mean Repetition Index (RI) of adult control zebrafish ±SEM (ns $p = 0.8187$, Kruskal-Wallis). EtOH (0.00006%) n = 4, EtOH (0.0015%) n = 19, EtOH (0.003%) n = 9. Values without a letter in common are statistically different to the control condition ($p < 0.05$, Kruskal-Wallis).

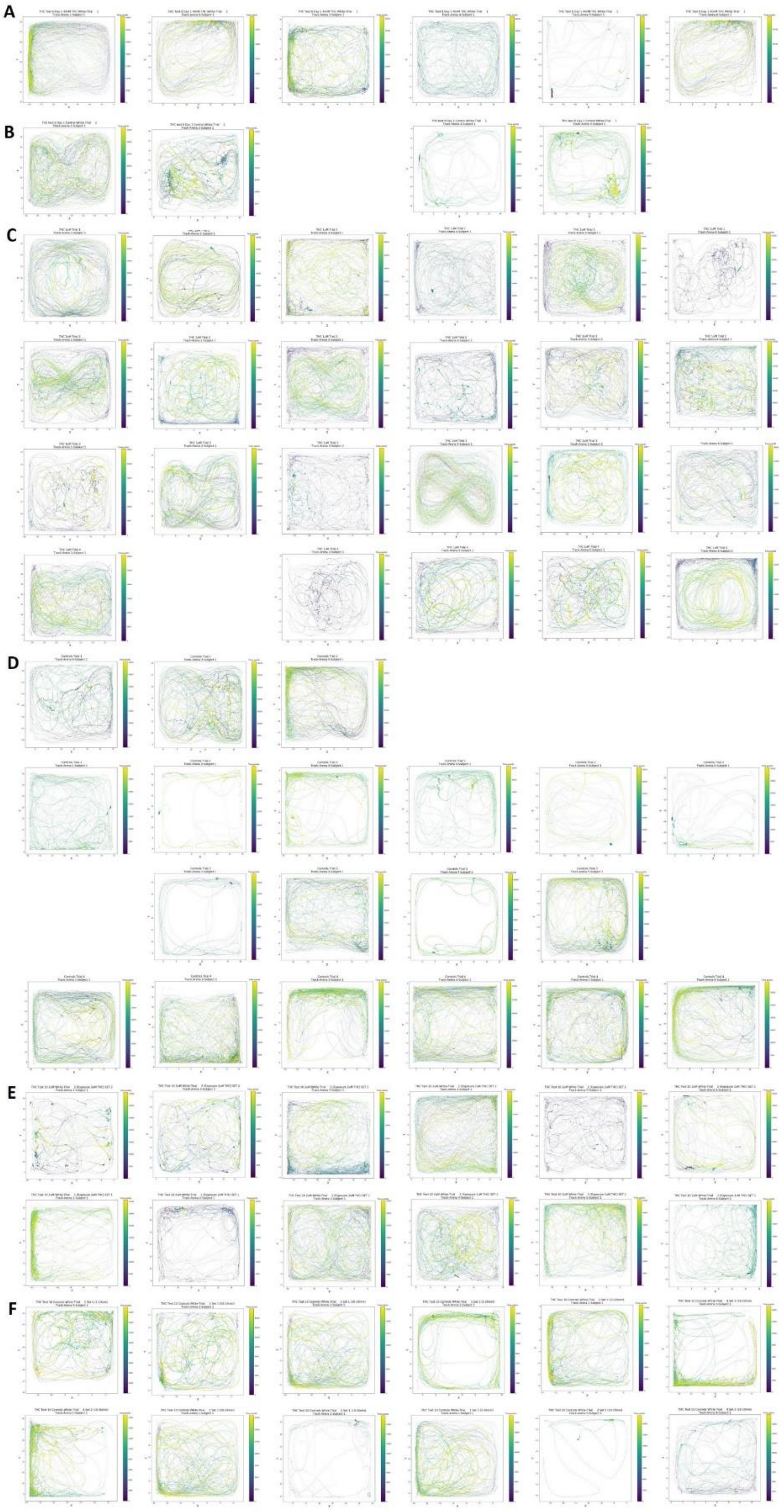

Figure S2. Raw tracking of adult zebrafish trajectory during 20-minute 40 nM **(A),** 1 µM **(C),** 2 µM **(E)** THC immersion and corresponding ethanol 0.00006% **(B),** 0.0015% **(D)** and 0.003% **(F)** controls.

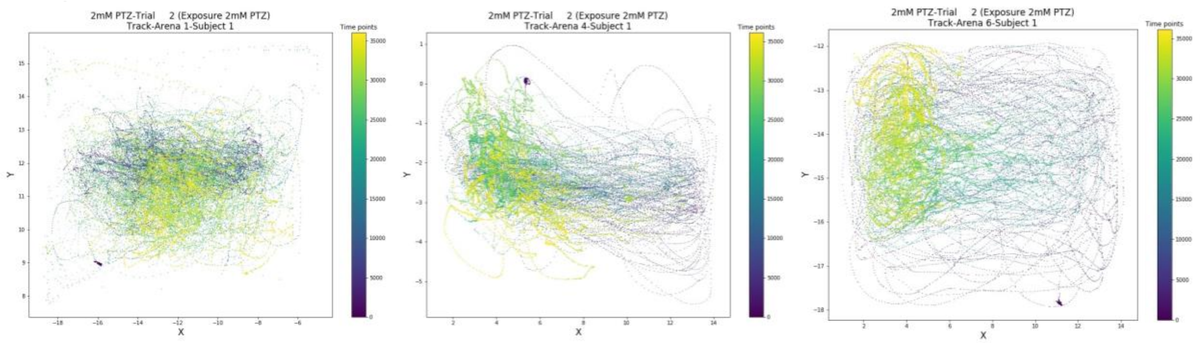

Figure S3. Representative movement tracks of convulsions evoked at 2mM PTZ.

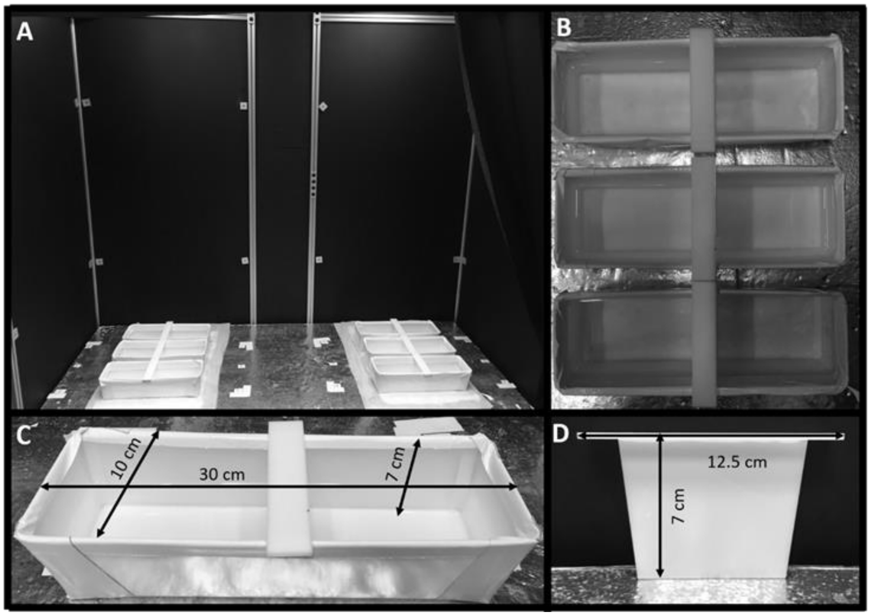

Figure S4. (A) A snapshot of the set-up for behavioural recordings. **(B)** Positioning of the testing tanks with partitions beneath the camera. **(C)** Dimensions of the white opaque testing tanks (30 cm (L) x 10 cm (W) x 7 cm (H)) and **(D)** dimensions of the white opaque partitions (12.5 cm (L) x 0.5 cm (W) x 7 cm (H)) dividing the testing tanks into two compartments.

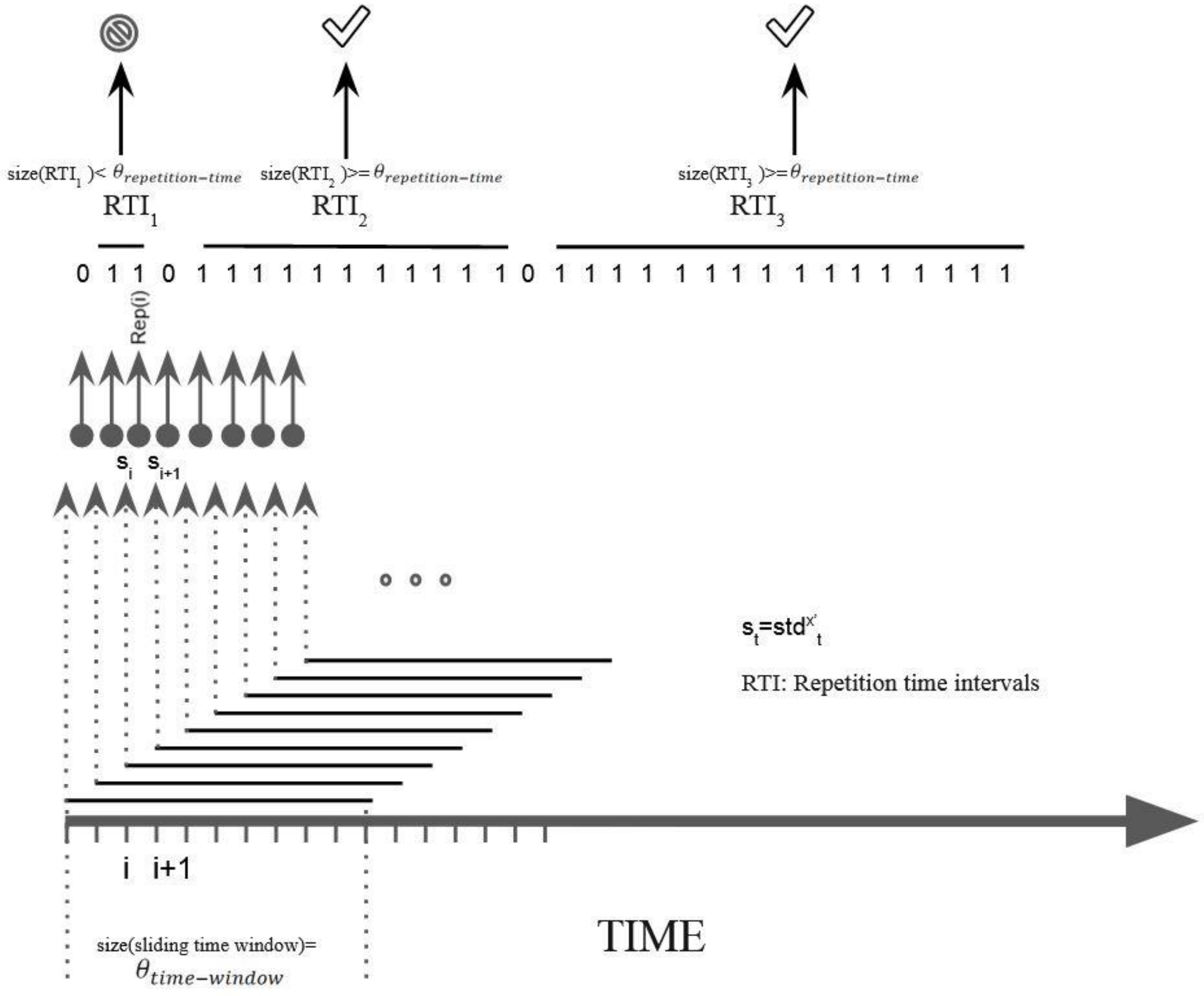

Figure S5. The raw x and y co-ordinates were used to calculate an unbiased Repetition Index (RI). Detected thigmotaxis movement near the edges of the tank was removed. If the standard deviation between the x and y co-ordinates within the specified window was below 0.01 ($<\theta_{std\text{-}change}$), the trajectory was considered repetitive and set to 1, If the standard deviation was above the threshold, the trajectory was considered arbitrary and set to 0. If the event set to 1 had a duration longer than 700 time points ($>\theta_{repetition\text{-}time}$), it was extracted as a cycle set, while shorter events ($<\theta_{repetition\text{-}time}$), were excluded. The durations of all cycle sets were summed, divided by the total time interval and normalized into RI values ranging between 0 to 1. Higher RI values signify intensified and prolonged repetitions in swimming trajectory, whereas values nearer 0 indicate more random movement.
